# Human DNA Virus Exploitation of the MAPK-ERK Cascade

**DOI:** 10.3390/ijms20143427

**Published:** 2019-07-12

**Authors:** Jeanne K. DuShane, Melissa S. Maginnis

**Affiliations:** 1 Department of Molecular and Biomedical Sciences, The University of Maine, Orono, ME 04401, USA; 2 Graduate School in Biomedical Sciences and Engineering, The University of Maine, Orono, ME 04401, USA

**Keywords:** mitogen-activated protein kinase, viruses, cellular signaling, infection

## Abstract

The extracellular signal-regulated kinases (ERKs) comprise a particular branch of the mitogen-activated protein kinase cascades (MAPK) that transmits extracellular signals into the intracellular environment to trigger cellular growth responses. Similar to other MAPK cascades, the MAPK-ERK pathway signals through three core kinases—Raf, MAPK/ERK kinase (MEK), and ERK—which drive the signaling mechanisms responsible for the induction of cellular responses from extracellular stimuli including differentiation, proliferation, and cellular survival. However, pathogens like DNA viruses alter MAPK-ERK signaling in order to access DNA replication machineries, induce a proliferative state in the cell, or even prevent cell death mechanisms in response to pathogen recognition. Differential utilization of this pathway by multiple DNA viruses highlights the dynamic nature of the MAPK-ERK pathway within the cell and the importance of its function in regulating a wide variety of cellular fates that ultimately influence viral infection and, in some cases, result in tumorigenesis.

## 1. Introduction

**Figure 1:**
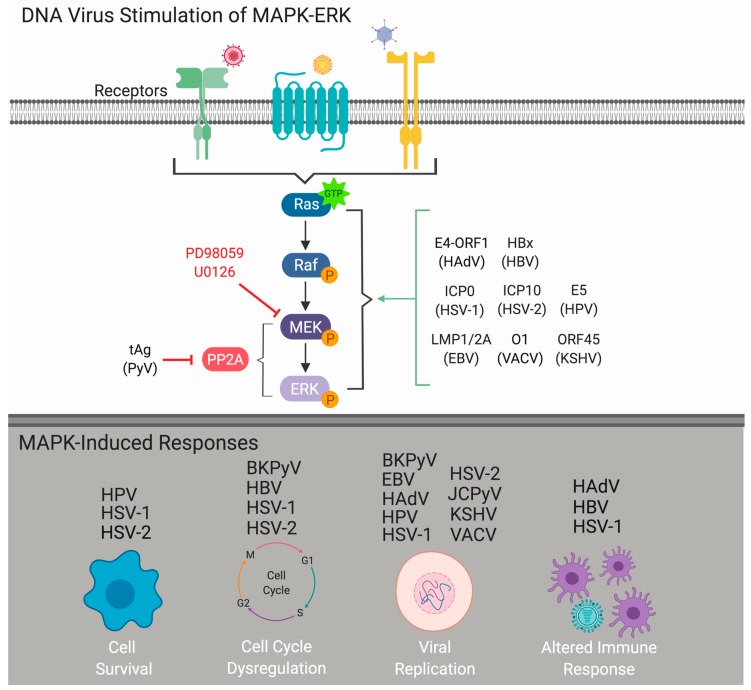
DNA virus stimulation of the mitogen-activated protein kinase extracellular signal-regulated kinase (MAPK-ERK) cascade. Viral stimulation of the MAPK-ERK pathway induces sequential phosphorylation of the core kinase of the MAPK-ERK signaling mechanism: Raf, MAPK/ERK kinase (MEK), and ERK. Upon activation of this pathway, multiple responses can be induced to help facilitate viral infection including promoting cell survival, cell cycle dysregulation, enhancement of viral replication, or alterations to host immune responses. Abbreviations: BK polyomavirus (BKPyV), Epstein-Barr virus (EBV), human adenovirus (HAdV), hepatitis B virus (HBV), human papillomavirus (HPV), herpes simplex virus 1 (HSV-1), herpes simplex virus 2 (HPV-2), JC polyomavirus (JCPyV), Kaposi’s sarcoma-associated virus (KSHV), vaccinia virus (VACV). Created with BioRender.

As obligate intracellular parasites, viruses require host cells for productive infection and replication. While the specific host-cell mechanisms required for successful infection vary between virus types, the induction or manipulation of host-cell signaling cascades is essential for successful viral infection and can influence viral pathogenesis. One pathway known to be required by many different types of DNA and RNA viruses is the extracellular signal-regulated kinase (ERK) cascade, a specific facet of the mitogen-activated protein kinase (MAPK) pathway (MAPK-ERK). As a central regulator of cellular response to environmental stimuli, the MAPK-ERK pathway predominantly transmits extracellular signals that induce cellular proliferation, differentiation, and survival [1]. Both DNA and RNA viruses usurp the MAPK-ERK signaling pathway to mediate multiple aspects of the virus infectious cycle [2,3,4]. DNA viruses alter MAPK-ERK signaling to promote viral internalization, dysregulate the cell cycle, regulate viral replication, and prevent host-cell death (Figure 1). Interestingly, the majority of DNA viruses traffic to the host-cell nucleus to carry out transcription and replication of the viral genome through the utilization of host-cell machinery. Thus, elucidating how DNA viruses regulate cell growth pathways such as the MAPK-ERK pathway is critical for a better understanding of viral infection and pathogenesis, including viral-induced cancers. This review is designed to provide an overview of how human DNA viruses exploit the MAPK-ERK signaling pathway to facilitate viral replication and subsequent pathogenesis.

## 2. MAPK-ERK Cascade

There are four distinct MAPK signaling cascades that play central roles in transmitting and interpreting extracellular stimuli into intracellular responses, including the extracellular signal-regulated kinase (ERK1/2), p38 MAPK, c-Jun N-terminal kinases (JNK1/2/3), and ERK5 [5]. The MAPK pathways are comprised of highly conserved serine/threonine kinases that are activated in response to external stimuli propagated through the phosphorylation of upstream MAP kinases [6]. These external stimuli can include hormones, growth factors, stress signals, or even invading pathogens that often interact with membrane-bound receptors to initiate the signaling cascade [7]. The MAPK-ERK pathway signals through three core kinases—Raf, MAPK/ERK kinase (MEK1/2), and ERK1/2—which are responsible for regulating a plethora of cellular responses including differentiation, proliferation, and survival [8]. Upon stimulation, the upstream MAP kinase kinase kinase (Raf) activates the MAP kinase kinase (MEK1/2), culminating in the phosphorylation of the MAP kinase (ERK1/2). The activation of this pathway can induce cellular growth and differentiation by means of upregulating host-cell DNA machineries downstream of phosphorylated ERK (pERK) [9]. Activated ERK1/2 proteins have been shown to interact with over 200 known cellular substrates, located in both the cytoplasm (RSK, FAK, MNK, etc.) and within the nucleus (Elk-1, c-Fos, c-Jun, etc.), giving this signaling pathway immense influence over the fate of the cell [10]. ERK1/2 regulates an array of nuclear transcription factors through phosphorylation, which in turn can regulate gene expression based upon the initial input stimulus. Moreover, these ERK-activated transcription factors like the AP-1 complex, can directly influence cyclins and thus cyclin-dependent kinases (CDKs) that regulate the cell cycle driving cells from G1 (gap 1, resting) to S (synthesis) phase [7,11]. On the flip side, the MAPK signaling cascade is further regulated by MAPK phosphatases (MKPs), which can act as negative or positive regulators of MAPK-ERK signaling. Protein phosphate 2A (PP2A) is essential as a negative regulator of the MAPK pathway through mechanisms including but not limited to the dephosphorylation of MEK or ERK [12]. Thus, by commandeering this canonical signaling mechanism, viral pathogens can utilize the MAPK-ERK cascade to fine tune the pathway for optimal viral replication.

Studies of the MAPK-ERK cascade within the field of virology, cellular biology, and biochemistry have made use of cell-based and biochemical assays to better define the utilization and molecular patterns of this pathway [13]. Cell-based assays are utilized to define the role of specific components of the MAPK pathways through treatment of cells with inhibitors and measuring infectivity or cell-signaling outcomes. Commercially available, highly selective inhibitors have made the study of the MAPK pathway in cultured cells relatively straightforward and reproducible. Specific inhibitors that have been widely utilized within the studies presented herein include PD98059 (2′-Amino-3′-methoxyflavone) and U0126 (1,4-diamino-2,3-dicyano-1,4-bis[2-aminophenylthio] butadiene), which both bind to MEK1 and MEK2, preventing ERK phosphorylation [14,15]. In addition to these inhibitors, the use of siRNAs to target Raf, ERK, or other components of the MAPK signaling pathway have provided added experimental advantages [4,13]. In addition to cell-based infectivity assays, biochemical assays to measure phosphorylation of the kinases within the MAPK cascade have been carried out using Western blotting or In-cell Western (ICW)^TM^ technologies [16]. Together, advances in experimental technologies have significantly increased our understanding of the MAPK pathway during the viral infectious cycle. However, this remains an important and active area of inquiry. Defining the signaling pathways utilized by viruses provides tremendous insight into virus–host cell interactions, shedding light on viral evolution, evasion of the host immune response, virus-induced cancers, and identifying potential antiviral therapeutics for pathogenic viruses.

## 3. DNA Viruses

### 3.1. Adenoviridae

#### Adenovirus

The human adenovirus (HAdV) family is comprised of over 60 different serotypes that infect a variety of human tissues [17]. While HAdV infections most commonly occur in the upper respiratory tract, they are also associated with a wide range of clinical diseases such as conjunctivitis, myocarditis, and gastroenteritis [17,18]. HAdVs are non-enveloped, double-stranded DNA viruses that range from 70–100 nm in diameter [19]. The genome of HAdVs, similar amongst all serotypes, is separated into early, intermediate, and late gene regions that parallel the infectious lifecycle [19]. The early region is divided into gene families E1–E4 that initiate viral replication processes. Intermediate transcripts IX and IVa2 are trailed by the late region, comprised of transcript families L1-L5, which facilitate virion maturation [17,19]. Interestingly, multiple groups have demonstrated that HAdVs require the induction of MAPKs like the ERK, p38, and JNK pathways to facilitate the completion of the viral replication process [20,21,22]. Early investigations into HAdV infection demonstrated that multiple serotypes of HAdV and HAd viral vectors activate MAPK-ERK signaling, facilitating both host-cell cytokine production and viral replication [23,24,25,26,27].

Much of the research regarding HAdVs has focused on its implementation as a delivery system for gene therapies, particularly focusing on elucidating the impacts of these HAdV vectors on host–immune responses such as the production of cytokines [28]. HAdVs induce the production of pro-inflammatory cytokines such as interleukin-8 (IL-8), which acts as a chemoattractant to recruit neutrophils and other immune cells to the site of infection [27]. HAd viral vectors and infectious wild-type HAdV activate the MAPK-ERK pathway upon viral attachment to induce the production of cytokine interleukin-8 (IL-8) [23,24,25]. The induction of IL-8 by HAdV-stimulated MAPK-ERK signaling occurs across multiple cell types and contributes to host-inflammatory responses during gene therapy administration of HAd viral vectors [23,24,25,28,29]. IL-8 induction poses a challenge for HAdV vector-mediated treatment delivery, yet suppression of IL-8 has been demonstrated to improve the efficacy of HAdV oncolytic vectors in cancer treatments [30]. However, it should also be noted that HAdV infection of polarized epithelial cells activates the production of IL-8 to upregulate the expression of the viral receptor coxsackievirus and adenovirus receptor (CAR), resulting in enhanced viral entry, suggesting that IL-8 production may enhance infection with CAR-utilizing HAdVs or vectors [31]. Furthermore, other studies have highlighted the role of HAd viral vector-induced MAPK-ERK signaling in the activation of microglia, the phagocytic immune cells in the CNS [32]. HAdV vectors stimulate the production of nitric oxide (NO) and inducible NO synthase (iNOS), which activate microglia and the host immune response [32]. Interestingly, NO and iNOS production is inhibited upon treatment with the MEK inhibitor PD98059, suggesting that inhibition of ERK activation prevents HAd vector-stimulation of inflammatory responses common in HAd vector gene therapy models [32]. These works, in particular, have helped to demonstrate the significance of the MAPK-ERK pathway in addressing the immunological consequences of HAd vector utilization for gene therapies.

More recently, HAdV research has focused on the impact of MAPK-ERK signaling on viral replication. While activation of MAPK-ERK occurs initially after HAdV challenge [23,26], ERK signaling facilitates only the later stages of viral infection [22,26]. ERK is activated in a transient and biphasic pattern in response to HAdV infection [23,26], suggesting that HAdV and HAd vectors induce MAPK-ERK signaling at various points in the viral lifecycle to promote multiple facets of the viral infection process. Inhibition of MEK via U0126 treatment reduces the production of viral progeny [26], ribosomal association with viral mRNA [26], as well as Ras-mediated HAdV replication [33]. Recently work has investigated the capacity of HAdV protein E4-ORF1, an activator of phosphatidylinositol 3-kinase (PI3K), to stimulate the MAPK-ERK pathway [34]. In particular, the authors identified the role of MAPK-ERK activation of the downstream transcription factor cMyc, which was shown to enhance HAdV replication [34]. The authors found that HAdV E4-ORF1 protein can promote the activation of MAPK-ERK through the epidermal growth factor receptor (EGFR) specifically to sustain cMyc levels in the nucleus [34]. Together, these findings suggest that HAdV targets activation of MAPK-ERK to facilitate the viral replication processes [22,26,34].

### 3.2. Poxviridae

#### Vaccinia Virus

Vaccinia virus (VACV), the prototypical member of *Poxviridae*, is a large, enveloped double-stranded DNA virus (360 × 270 × 250 nm in diameter) that is amongst the most complex of mammalian viruses. With a genome of ~200 kpb in length, VACV encodes for over 200 genes [4,35], including viral-dependent polymerases, which enable the virus to complete the replication process solely within the cytoplasm of the infect host cell. VACV, a close evolutionary relative of variola virus, the cause of smallpox, is utilized in the current smallpox vaccination regimen [36]. Due to the live vaccinia virus formulation of the vaccine, related complications after exposure include accidental inoculation, the development of eczema vaccinatum or progressive vaccinia, and myocarditis [36,37]. However, wide-spread vaccination was halted in the 1970s due to worldwide smallpox eradication, and vaccination is now limited to laboratory staff and military personnel due to potential bioterror threats [36,38].

VACV infection results in the activation of the MAPK-ERK pathway and the subsequently produced viral gene products drive sustained MAPK signaling to facilitate viral replication [4]. Over the course of the infectious process, VACV-infected cells secrete a polypeptide, VACV-induced growth factor (VGF), that competes for binding to the epidermal growth factor receptor (EGFR) on the cell surface [39]. Interestingly, prototypical EGFR signaling stimulates mitogenic processes like cellular growth and proliferation, often through canonical MAPK-ERK signaling [40]. The utilization of EGFR during infection suggests that VACV could use the mitogenic signaling potential of EGFR to activate the MAPK-ERK cascade, yet VGF is not required for sustained ERK1/2 activation [41]. Interestingly, the early activation of the MAPK-ERK pathway facilitates the expression of the viral early gene thymidine kinase (TK), an enzyme critical for viral replication [42]. Experiments employing the MEK inhibitor PD98059, demonstrated delayed TK expression and a significant decrease in infectious VACV progeny production, highlighting the role of MEK and ERK in VACV replication [41,43]. Moreover, VACV promotes sustained activation of ERK1/2 through the VACV O1 protein [41,44] and the downstream target ribosomal S6 kinase 2 (RSK2) in a multiplicity of infection (MOI)-dependent fashion, suggesting enhanced MAPK-ERK facilitates multiple stages of the viral lifecycle [41,44]. For example, VACV-induced MAPK-ERK activation has downstream impacts on viral replication. Transcription factor targets of phosphorylated ERK (pERK) such as cFos [4], early growth response 1 (EGR-1) [45], and c-Jun [46], are temporally regulated by VACV during the infectious lifecycle through direct modulation of MAPK-ERK in order to dysregulate host-cell functions and promote viral replication [4,45,46]. In sum, VACV activates the MAPK-ERK pathway upon initial infection to promote the expression of viral proteins like O1 leading to sustained MAPK-ERK activation, which is necessary for viral replication. These findings demonstrate the necessity for highly orchestrated MAPK-ERK activation in VACV infection.

### 3.3. Polyomaviridae

In recent years, discovery of human polyomaviruses has expanded greatly, with the identification of over 10 new viral species [47,48,49,50,51,52,53,54,55,56,57]. With such rapid discovery of new polyomaviruses, it is critical that investigations into their potential impacts on human health keep pace. Polyomaviruses have differing tissue tropisms, yet generally only cause diseases in the immunocompromised [58,59]. Clinical manifestations of polyomavirus infections include a wide range of illnesses such as skin cancer, polyomavirus-induced nephropathy (PVN), and demyelinating disease of the central nervous system (CNS) [58,60,61,62]. Polyomaviruses are icosahedral, non-enveloped viruses that are approximately 40–45 nm in diameter [63]. Each has a circular double-stranded DNA genome that are ~5200 bp in size [63]. The overall structure of the genome is divided into two main sections – early and late viral genes separated by a non-coding control region (NCCR). The viral early genes (T-antigens) and late genes (viral proteins 1, 2 and 3 and agnoprotein), are temporally regulated to support productive infection within the host [58,63]. Encoded within the early region are small t-antigen (tAg), large T-antigen (TAg) and an alternatively spliced large T-antigen or middle t-antigen (Tag) [63,64]. Of these, TAg plays a crucial role in promoting polyomavirus replication through varying biological activities within the cell that help to facilitate the production of the late viral proteins VP1-3, which comprise the viral capsid [63,64]. Despite the disparity in tissue tropism and disease manifestations, members of the *Polyomaviridae* converge at the utilization of the MAPK signaling pathway during infection. Interestingly, in addition to activation of the MAPK pathway, a number of polyomaviruses encode for proteins that specifically bind to the protein phosphatases and thus can regulate the activation of the MAPK within the host cell for viral replication.

#### 3.3.1. JC Polyomavirus

The human pathogen JC polyomavirus (JCPyV) was discovered in 1971 when it was isolated from the brain of an individual with progressive multifocal leukoencephalopathy (PML) [65]. JCPyV is projected to infect between 50–80% of the human population, causing a persistent asymptomatic infection in the kidney of healthy individuals [66,67]. In humans, JCPyV is the etiological agent of PML, a fatal neurodegenerative disease characterized by the viral lysis of glial cells, astrocytes and oligodendrocytes within the CNS [68,69]. Resultant PML lesions are caused by the destruction of the myelin-producing glial cells leading to a loss of the myelin sheath on neuronal axons [70]. These lesions are typically multifocal across varying regions of the brain suggesting that the spread of the virus into the CNS is hematogenous in nature [70]. Since the onset of the HIV epidemic in the 1980s [71], the majority of PML cases have been linked to HIV+ individuals. As of 2005, nearly 80% of reported PML diagnoses have been associated with HIV infection [72].

Research into the infectious process of JCPyV has predominantly been focused on the viral attachment, internalization, and trafficking mechanisms necessary for infection. However, recent studies have begun to focus on the importance of viral manipulation of cellular signaling cascades, including MAPK-ERK, that facilitate these infectious processes. Signaling mechanisms like MAPK-ERK transmit extracellular stimuli inward toward downstream cellular targets, often through membrane-bound proteinaceous receptors. In the case of JCPyV, viral internalization requires the receptors of the 5-hydroxytryptamine subtype 2 family (5-HT_2_R) [73], receptors that have been directly linked to the activation of the MAPK-ERK pathway [74]. Although a direct link between JCPyV and 5-HT_2_R has not yet been established, ERK becomes phosphorylated in JCPyV-infected cells at timepoints coinciding with viral entry, suggesting JCPyV activates the MAPK-ERK pathway early during infection [75,76]. JCPyV-induced ERK phosphorylation is multiphasic in nature, demonstrating transient activation patterns that appear to facilitate multiple phases of viral infection [75,76]. However, early activation of ERK is not required for viral attachment or entry processes, indicating that while JCPyV-induced MAPK-ERK signaling occurs early on, this pathway plays a role in promoting the later stages of infection such as viral transcription and overall viral replication [75,76]. Moreover, JCPyV infection has been shown to require receptor tyrosine kinase (RTK) activity, which may highlight alternate mechanisms for MAPK-ERK activation during JCPyV infection [75,77].

Multiple reports have indicated that MAPK-ERK activation during JCPyV challenge is required for successful infection in multiple cell types [75,76,78]. Chemical inhibition of MAPK-ERK proteins, including Raf (Bay43-9006) and MEK (PD98059, U0126), results in significant reductions in JCPyV infectivity [16,75,76,78]. This inhibition of ERK activity may play a direct role in preventing ERK nuclear localization and subsequent activation of the requisite transcription factors needed to facilitate viral replication. Treatment of glial cells in vitro with ERK siRNA causes a significant reduction in viral early gene T-antigen transcripts, suggesting that ERK activity plays a role in facilitating the transcription of viral gene products [76]. Interestingly, protein phosphatase 2 (PP2A), which is known to dephosphorylate members of MAPK-ERK, physically interacts with JCPyV tAg, which may allow for sustained activation to these core kinases during infection [79]. Seemingly, JCPyV activation of MAPK-ERK involves the complex regulation of multiple proteins within and adjacent to the MAPK-ERK pathway in order to promote dysregulation of canonical signaling and promote viral replication.

Early studies of JCPyV infection characterized transcription factors that are necessary for viral replication of early and late genes including SMAD4, cMyc, c-Jun, cFos (AP1), and nuclear factor of activated T-cells (NFAT4) [80,81,82,83,84,85]. Interestingly, many of these identified host-factors are downstream targets of the activated MAPK-ERK pathway [1]. Previous work has demonstrated that an activator of the transcription factor SMAD4, TGF-β1, stimulates JCPyV replication by increasing SMAD association with JCPyV promoter sequences [78]. This stimulation, however, was blocked by the MEK inhibitors PD98059 and U0126, suggesting that TGF-β1 stimulation of the JCPyV replication process occurs through the MAPK-ERK pathway [78]. Expression of the early viral gene product TAg has been shown to increase NFAT4 activity in glial cells [83], suggesting that the PKC-calcium corridor, an activator of MAPK-ERK, may also play a role in JCPyV infection. Taken together, these findings suggest that JCPyV activation of the MAPK pathway may regulate the activation of transcription factors necessary for viral infection.

#### 3.3.2. BK Polyomavirus

BK polyomavirus (BKPyV) is the causative agent of polyomavirus-associated nephropathy and hemorrhagic cystitis in humans, most often associated with complications resulting from kidney transplants [86]. This virus was first identified in 1971, from a renal transplant recipient who presented with a case of ureteric stenosis [87]. The majority of infected individuals acquire a primary BKPyV infection during childhood, resulting in a lifelong, persistent infection, similar to that seen during JCPyV infection [67]. Interestingly, this closely related polyomavirus shares 75% sequence homology with JCPyV [88,89], potentially contributing to similar patterns of transmission, patterns of persistence, and opportunistic induction of disease states in immunocompromised individuals between polyomaviruses [88,90].

Similar to JCPyV, expression of BKPyV T-antigen proteins modulates host-cell activity in order to reprogram host-cell functions towards generating new viral progeny. BKPyV small t-antigen binds to PP2A, thus preventing the phosphatase from regulating the MAPK-ERK cascade and stimulating proliferation [91,92,93]. Moreover, BKPyV replication is enhanced through MAPK-ERK signal activation, and chemical inhibition of this pathway with the MEK inhibitor U0126 decreases infection, suggesting that this virus utilizes the MAPK-ERK pathway specifically to facilitate host-cell infection [94]. Interestingly, BKPyV activation of ERK1/2 also promotes increases in cyclin D1 levels, a key regulator of the cell cycle [94]. Together these findings suggest that polyomaviruses impact the MAPK-ERK pathway to control host cell cycle progression to facilitate viral replication.

#### 3.3.3. Other Polyomaviruses

Interestingly, a number of human polyomaviruses that are associated with specific pathologies of the skin have been shown to activate or utilize the MAPK cascade resulting in activation of the transcription factor c-Jun. For instance, human polyomavirus 6 (HPyV6), which infects keratinocytes, is chronically shed from the skin and has recently been associated with clinical manifestations of the skin including pruritic and dyskeratotic dermatoses [95], which activates the MAPK cascade during infection [96]. HPyV6 induces MEK-ERK phosphorylation through small T-antigen binding to PP2A, leading to the upregulation of c-Jun [96]. Activation of MAPK-induced c-Jun is implicated in a number of cancers and thus highlights a potential role for HPyV6 in contributions to squamous cell carcinomas [96]. Furthermore, Trichodysplasia Spinulosa (TS) polyomavirus (TSPyV), which causes TS, a rare condition of the skin that leads to disfigurement [97], encodes for a small T-antigen that leads to hyperactivation of MEK and ERK and phosphorylation of c-Jun [98]. TSPyV small T-antigen and middle T-antigen bind to PP2A [95,99], and it is likely that this interaction prevents PP2A from dephosphorylating kinase targets of the MAPK signaling pathway. Overall, TSPyV activation of the MAPK pathway and resultant c-Jun phosphorylation likely contributes to cellular proliferation and development of TSPyV lesions [98]. On the other hand, Merkel cell polyomavirus (MCPyV), which can cause Merkel cell carcinoma, a rare but aggressive form of skin cancer, also upregulates c-Jun phosphorylation but does not activate MEK or ERK [100]. In summary, the activation of the MAPK pathways by polyomaviruses can result in upregulation of c-Jun contributing to proliferative skin pathologies.

### 3.4. Papillomaviridae

#### Human Papillomaviruses

Human papillomaviruses (HPV) of the *Papillomaviridae* family are small, non-enveloped, double-stranded DNA viruses, most often associated with cervical cancer development [101,102]. The typical HPV virion is ~60 nm in diameter encasing a circular, double-stranded DNA genome of ~8 kb that encodes for eight proteins [101,102]. HPVs predominantly infect either cutaneous or mucosal surfaces, which leads to the development of a variety of skin pathologies and cancers [103]. HPV infection of cutaneous regions can cause warts including common and plantar warts as well as rare forms of skin cancer [103]. Infection of mucosal surfaces results in disease pathologies more commonly associated with HPV infection including condyloma acuminatum, cervical cancer, and head and neck cancers [103]. Remarkably, there are more than 200 identified papillomaviruses [103,104], further classified into low-risk or high-risk HPV types based on their association with cancer development [103,104]. There are 14 or more high-risk types of HPV that can cause abnormal cellular changes to the cervix, yet types 16 and 18 are responsible for ~70% of cervical cancers [105] and are the most prevalent HPV types worldwide [106]. Due to their high prevalence and link to human cancers, the high-risk HPV types have been widely studied to define mechanisms of pathogenicity and carcinogenesis.

Cancer development during HPV infection has been strongly linked to the overexpression of the viral early genes E6 and E7 [107]. These viral oncogenes promote the dysregulation of the cell-cycle, prevention of apoptosis, and the initiation of cell proliferation mechanisms, roles central to productive HPV infection and the resultant development of a variety of cancers [108]. HPV E7 targets numerous cell cycle control proteins for degradation, including the retinoblastoma (Rb) protein family, leading to cellular proliferation, while E6 targets the tumor suppressor p53 for degradation and thus promotes immortalization and cellular transformation [109]. The degradation of p53 occurs upon E6 binding and involves the utilization of the ubiquitin-dependent protease system, leading to a significant loss of tumor suppression signaling in HPV infected cells [110]. In addition, HPV16 E6 and E7 interact with the MAPK-ERK pathway during infection [111,112,113,114]. Previous reports have identified that in vitro overexpression of the HPV16 E6 and E7 proteins resulted in prolonged levels of enhanced ERK phosphorylation during HPV infection, which was subsequently abrogated upon chemical inhibition of MEK with U0126 treatment [115,116,117]. Interestingly, the development of HPV-induced cancers has also been linked to the upregulation of two key mitogenic pathways including EGFR signaling and the stimulation of vascular endothelial growth factor (VEGF) by the multifunctional viral protein E5 [118,119,120,121,122]. HPV16 E5 upregulates VEGF expression through the activation of ERK, an effect dampened upon chemical inhibition of ERK phosphorylation with U0126 [120]. In addition to the upregulation of VEGF, E5 stabilizes EGFR leading to an enhancement of MAPK-ERK signaling [123]. Additional works have implicated the E5 protein in providing protection from apoptosis and autophagy in HPV-infected cells through the utilization of the MAPK-ERK cascade [124,125,126]. The overexpression and enhanced stimulation of EGFR upon HPV infection has also been well-documented and has been shown to result in the phosphorylation of ERK [123,127,128,129]. Further, HPV utilization of the EGF receptor is required for HPV E6 splicing, and this process is dependent on ERK signaling, as treatment with U0126 or overexpression of a dominant-negative MEK1 resulted in enhanced E6 exon exclusion [130]. Ultimately, these findings demonstrate the role of MAPK-ERK in promoting critical steps in HPV infection including host-cell survival, which can ultimately result in the development of HPV-induced human cancers.

### 3.5. Herpesviridae

*Herpesviridae* comprise a large family of viruses with nine virus species that routinely infect humans. They are characterized as large enveloped double-stranded DNA viruses with a virion diameter ranging from 100–300 nm with a unique and complex icosahedral structure [131]. The unique four-layered structure includes an envelope decorated with viral glycoproteins underlaid by a tegument layer, a nucleocapsid, and a core, which houses the viral genome. Linear herpesvirus genomes range in size from 125–240 kbp with ~70–170 protein-coding genes [132]. Gene expression is temporally regulated during the infectious cycle, and thus the viral genes are appropriately deemed: immediate early (IE), early, and late genes [133]. The IE genes are expressed immediately upon infection and are necessary to activate the early genes, which drive DNA replication, and the late genes, which provide the structural components. A further defining feature of herpesviruses is that, in addition to the lytic replication cycle, they can establish latency within the host with periodic episodes of reactivation [133]. Due to the large size and variation in herpesviruses genomes, it is not surprising that this family of viruses has a broad tissue tropism and can cause a wide range of outcomes in the human host. Thus, herpesviruses are organized into three subfamilies—*alpha*, *beta*, and *gamma*—based on their sizes and sites of latency. *Alphaherpesvirues* generally infect epidermal cells and establish latency in the sensory neurons. *Betaherpesviruses* have a broad tropism for tissues including lymphocytes, secretory glands, and the kidney. Finally, *gammaherpesviruses* have the narrowest host range, replicating in lymphoblast B and T cells [134]. Despite the broad range in virion and genome size, tissue tropism, and disease outcomes, a number of herpesviruses utilize the MAPK-ERK pathway to facilitate a successful viral infection.

#### 3.5.1. Herpes Simplex Virus

Herpes simplex virus 1 and 2 (HSVs) are members of the *Alphaherpesvirinae*, defined by relatively short infectious cycles and dynamic replication pattern [134,135]. The enveloped virions of HSV-1 and HSV-2 are ~200 nm in diameter [136,137]. HSVs cause oral and genital lesions, and, in rare cases, viral encephalitis [138]. The seroprevalence of HSV-1 across the globe is estimated to be ~90% [139], significantly higher than that of HSV-2 [140]. Some human herpesviruses are both oncogenic (tumor forming) and oncomodulatory (modulate tumor behavior), due in large part to the establishment of latency within the host cell [141]. Upon exposure, HSVs will induce latent infections in the neuronal sensory ganglia, maintained throughout the lifetime of the host, with periodic cycles of lytic reactivation [135].

The HSV-1 and HSV-2 genomes comprise over 70 distinct protein-coding genes that encode for proteins such as glycoprotein B (gB) and the infected cell proteins (ICPx) [131]. Interestingly, reactivation of HSV-1 into a lytic state requires the expression of IE genes within infected cells [142]. The accumulation of IE gene products drives viral early gene transcription and subsequent replication, resulting in the lytic infectious program. The complexity of the HSV-1 infectious lifecycle can be appreciated through the examination of HSV-1-induction of the MAPK-ERK pathway. Dysregulation and manipulation of MAPK-ERK signaling cascade facilitates multiple steps in the viral lifecycle including dampening of the host-cell anti-viral response, viral replication, and cell cycle control [141,143,144,145].

In order to facilitate infection, HSV-1 temporally regulates gene expression, which influences a spatial and progressive regulation of specific cellular proteins. Within the first 15 minutes of HSV-1 challenge, levels of pERK significantly increase, which are predominantly localized to the cytoplasm of the infected cell [145]. However, at later stages of infection congruent with viral replication, both pERK and ICP0, a multifunctional IE gene, localize to the nucleus, suggesting that ICP0 function requires ERK activity [145]. ICP0 function has predominantly been associated with cell cycle regulation within cells infected with HSV-1, through cyclin D3 [146,147]. Through the implementation of dominant-negative ERK1 constructs, Colao et al. demonstrated that ERK1 activity is necessary for cell cycle control during HSV-1 infection, which is in turn required for viral replication [145]. Interestingly, HSV-1 infection also requires cyclin E and the cyclin-dependent kinases (CDK) 2 and 5 for infection, suggesting further viral influence over the cell cycle and viral replication [145,148,149]. HSV-2 also utilizes the MAPK-ERK pathway to dysregulate the cell cycle, specifically through ribonucleotide reductase R1 protein (ICP10), to induce a proliferative cellular state [141,150,151], demonstrating the conserved utilization of the MAPK-ERK pathway for cell cycle dysregulation between these human herpesviruses.

Antiviral immune responses activated upon infection are important mechanisms that allow the host to recognize foreign pathogens. Activation of pattern-recognition receptors like toll-like receptors (TLRs) initiate the production of the antiviral signaling protein interferon (IFN), which is secreted from infected cells. IFN binding to IFN receptors on the same or neighboring cells, activates the IFN signaling pathway resulting in activation of IFN-stimulated genes such as the protein kinase regulated by dsRNA (PKR). PKR phosphorylates eIF2α resulting in halted protein translation, which is an effective way of protecting the cell from production of viral proteins. Productive HSV-1 infection requires the inhibition of PKR, via viral protein ICP34.5 and MAPK-ERK activity, which prevents the phosphorylation and subsequent deactivation of the eIF2α complex [141,143,144]. Inhibition of MEK with the chemical inhibitor PD98059 resulted in decreased levels of pERK, increased levels of PKR phosphorylation, and subsequent loss of late viral protein accumulation [152]. Additional studies have identified regulation of cellular anti-apoptotic activity during HSV infection via the viral gene US3 [153,154,155,156], a protein that has been shown to inhibit the MAPK-ERK pathway [156]. Conversely, the large subunit of HSV-2 ribonucleotide reductase, ICP10, induces ERK activity in order to block JNK-stimulated apoptotic signaling in hippocampal neurons [157]. Through the activation of Bag-1, ICP10-induced ERK is able to override these cell death signals, indicating a pro-survival implementation of the MAPK-ERK cascade during HSV-2 infection [157]. These broad mechanisms highlight multidimensional applications of the MAPK-ERK pathway utilized by HSV proteins.

Beyond regulation of the cell cycle and pro-survival signaling, the MAPK-ERK pathway also facilitates the process of viral replication. During HSV-2 infection, ERK activation occurs during the early steps of viral infection and is sustained through 24 hours post infection [152,158]. When phosphorylation of ERK is inhibited by U0126 or MEK1/2 siRNA treatment, HSV-2 propagation is decreased as measured by the production of viral proteins UL30 and gB [158]. These findings suggest that HSV-2 upregulates MAPK-ERK signaling to promote viral replication [158]. HSV-2 infection specifically activates components of the MAPK-ERK pathway including Ras, ERK, and the downstream transcription factor target, cFos [159], indicating that HSVs dysregulate MAPK-ERK to dampen the immune response, dysregulate the cell cycle and induce pro-survival signaling, but can also utilize this cascade to facilitate replication of the viral genome.

#### 3.5.2. Kaposi’s Sarcoma-Associated Herpesvirus

The oncogenic Kaposi’s sarcoma-associated herpes virus (KSHV), of the *Gammaherpesvirinae* subfamily, was first identified in 1994 [160]. Associated with the disease from which the name was derived, Kaposi’s sarcoma (KS) results in soft tissue tumors and has also been linked to the development of primary effusion lymphoma (PEL) [161]. KSHV has a genome of ~170 kbp in length, which encodes for a variety of latent, early lytic, and late lytic proteins [162]. KSHV DNA is present in all KS tumors often in conjunction with HIV, however, this is not a prerequisite for KS tumor formation [163]. Like other herpesviruses, KSHV adopts either a latent or lytic gene expression pattern, with lytic replication induced by the expression of the replication and transcription activator (RTA) viral protein [163]. Interestingly, activation of RTA can be induced in vitro through chemical application of phorbol esters like TPA (12-*O*-tetradecanoyl-phorbol-13-acetate) [164,165,166,167,168,169]. TPA is also associated with direct activation of the MAPK-ERK signaling pathway [164], potentially linking MAPK-ERK signaling with KSHV entrance into the lytic replication phase [165,168].

A key member of the MAPK-ERK pathway, Raf, was identified as playing a critical role in promoting KSHV infection [170]. Interestingly, inhibition of MAPK-ERK signaling through treatment with PD98059 and U0126, as well as bRaf siRNA treatment demonstrated that inhibition of MAPK-ERK signaling prevented KSHV reactivation [165,166,168,171,172]. Activation of MAPK-ERK signaling occurs early in the infectious cycle, within minutes of viral challenge, but is not required for viral attachment or internalization [171,172,173], similar to the activation pattern of the aforementioned JCPyV [75,76]. Increased MEK and ERK activity also plays a role in facilitating transcription of viral genes such as ORF8, ORF50, ORF73, and gB [168,173] upon nuclear localization of ERK [173]. The activation of MAPK-ERK during infection does increase RTA activity [171], but is also linked to KSHV utilization of transcription factors downstream of MAPK-ERK, such as the c-Jun, cFos, and cMyc [171,173,174]. The immediate early lytic protein ORF45, binds to and activates RSK, preventing the dephosphorylation of both ERK and RSK [174,175,176]. Cumulatively, these findings demonstrate that KSHV infection drives MAPK-ERK signaling to promote the reactivation from latency to promote the production of infectious progeny.

#### 3.5.3. Epstein-Barr Virus

Epstein-Barr virus (EBV), the prototype member of the *Gammaherpesvirinae* subfamily, infects ~90% of the world human population [177]. EBV, most commonly associated with mononucleosis, was first isolated in 1964 from a case of Burkitt lymphoma [178]. More recently, EBV has been associated with other malignancies including nasopharyngeal carcinoma (NPC), as well as non-Hodgkin’s and Hodgkin’s lymphomas [177]. EBV infects memory B cells, a viral tactic to evade the host-immune system, that helps in facilitating the development of a latent infection [179,180]. The EBV genome encodes for ~80 genes related to both latent and lytic infectious cycles [181]. The establishment and maintenance of the latent infection requires the expression of EBV latency genes such as the latent membrane proteins (LMP) LMP1, LMP2A, and LMP2B [181].

The latent infection of primary B cells by EBV and subsequent cellular transformation requires activation of MAPK-ERK signaling by LMP1 [182]. ERK activity is induced by LMP1, mediated through Ras signaling [182,183]. EBV-induced activation of MEK and ERK plays a role in increasing EBV infectivity as well as facilitating the reactivation of latent EBV [184]. Inhibition of proteins within the MAPK-ERK pathway have been shown to decrease EBV infectivity as well as induce a negative effect on EBV-infected B cell viability [185]. LMP1 plays a significant role in promoting epithelial cell invasion, an oncogenic property that may contribute to EBV-induced tumor formation. Interestingly, the ability of LMP1 to promote cell invasion and motility requires the MAPK-ERK pathway, suggesting that EBV manipulation of this signaling pathway not only promotes infection through downstream activation of transcription factors like the AP-1 complex but may also promote viral pathogenesis within the host [186,187]. LMP2A is another EBV latency protein that impacts the MAPK-ERK pathway to promote infection. Previous research has identified that this protein both phosphorylates and is phosphorylated by ERK [188,189,190]. These activation patterns may suggest that ERK activation of LMP2A may be necessary to facilitate LMP2A-mediated cell migration, or that LMP2A activates ERK in order to influence downstream transcription factor targets like c-Jun [189]. Both LMP1 and LMP2A are EBV proteins that help to maintain viral latency, typically in primary B lymphocytes [182,190]. Through altering mechanisms of MAPK-ERK activation, these proteins may be required for facilitating multiple facets of the latent infection within the B cell microenvironment.

### 3.6. Hepadnaviridae

#### Hepatitis B Virus

Hepatitis B virus (HBV) is the prototype member of the *Hepadnavirus* family and named for causation of hepatitis [191]. Chronic HBV infection is associated with cirrhosis of the liver and hepatocellular carcinoma [191,192,193,194]. The intact enveloped virion is ~42 nm in diameter [195] and contains a gapped, partially double-stranded DNA genome of ~3000 bp in length. Interestingly, the HBV genome encodes an encapsidated DNA polymerase, that is covalently linked to the viral genome, one of the unique and defining features of *hepadnaviridae* [196,197]. The HBV genome encodes for seven proteins: preCore, Core, pol, HBx, and the envelope proteins L, M, and S. HBx, a multifunctional regulatory transactivating protein, targets a variety of cellular substrates to induce cell cycle progression [3,198] including the MAPK-ERK pathway. HBx plays an essential role in the development of hepatocellular carcinoma (HCC), a disease most closely associated with chronic HBV infection [195,199,200]. While the HBx protein plays many different roles over the course of infection, it is involved in the dysregulation of signaling mechanisms such as MAPK-ERK to promote several different stages of HBV infection [3,200]. HBx directly activates the MAPK-ERK pathway [201] through the activation of Ras, leading to cell progression into the S phase [200] initiating a proliferative cellular state through the upregulation of cyclin D1 [201]. While HBx does not directly phosphorylate ERK, it does activate a transcription factor target of MAPK-ERK signaling, c-Jun [202]. Moreover, the middle surface antigen (MHBs(t)) has been shown to upregulate activation of MAPK-ERK through PKC-dependent signaling in order to dysregulate the cell cycle [203].

Recently, the HBV core antigen protein (HBcAg) has been shown to induce IL-6 cytokine production through the activation of the MAPK-ERK pathway in hepatocytes [204]. The activation of this cytokine is blocked during chemical treatment with the MAPK inhibitor U0126, suggesting that MAPK-ERK signaling facilitates HBcAg induction of IL-6 [204]. As IL-6 plays an integral role in the development of hepatocyte inflammation, liver disease, and liver cancers, the utilization of the MAPK-ERK during HBV infection may highlight a potential role of this pathway facilitating the development of HCC and other liver cancers in vivo. Indeed, within the mouse model, the multifunctional HBx protein activates ERK, which can be ablated during treatment with the MEK inhibitor PD98059 [205]. This ERK activation is sustained in vivo for nearly 30 days, resulting in persistent AP-1 complex activation [205]. These findings suggest that multiple HBV proteins target the MAPK-ERK pathway to alter cell cycle progression and increase the activity of specific MAPK-induced transcription factors.

## 4. Conclusions

The interplay between virus and host is an exceedingly complex and strategic battle, the consequences of which can have long-lasting and broad implications in the host. A successful viral infection is predicated on viral manipulation and reprogramming of key host-cell proteins that will facilitate replication of the viral genome. As a master regulator of key processes within the cell, the MAPK-ERK cascade plays a critical role in promoting gene expression, activating pro-survival mechanisms, inducing host-cell immune mediators, and regulating the cell cycle [1]. Each of these mechanisms are also utilized by the viruses described herein. By hijacking the MAPK-ERK pathway, viruses have near unlimited control over the fate of the cell, allowing viruses to manipulate cells for viral replication (Table 1).

The regulation of the MAPK-ERK pathway by DNA viruses is dynamic in nature, highlighted by both the similar and differential utilization of this pathway by closely-related viruses. As described, multiple members of the *Polyomaviridae* family of viruses stimulate MAPK-ERK activation through viral disruption of MAPK-ERK regulators like PP2A, to facilitate viral genome transcription and replication [79,91,92,93]. However, HSV-1 activates MAPK-ERK to promote changes to the cell cycle to induce a proliferative state, while HSV-2 employs MAPK-ERK activity to suppress apoptotic signaling to ensure cell survival [145,157]. MAPK-ERK plays an integral role in many different biological processes, and it is the sheer complexity of this interconnected signaling network that allows for a multitude of cellular endgames for a virus to exploit, many of which have significant consequences on the overall health of the host.

There is a conspicuous link between viruses and the development of human cancers, of which an estimated 20% of reported cases are caused by viral infection [206,207]. In particular, the vast majority of these virus-induced cancer cases are caused by seven distinct viruses, five of which are DNA viruses—EBV, KSHV, HPV, HBV, and MCPyV. However, this seemingly unintended consequence of cancer development due to virus infection may be directly linked to viral dysregulation of the MAPK-ERK pathway. As aberrant Raf activity occurs in nearly 10% of all cancers [208], and the MAPK-ERK pathway is dysregulated by a host of DNA viruses, the MAPK-ERK cascade is a promising target for facilitating viral replication that may also play a role in DNA viral-induced tumor formation. While not all DNA viruses are definitively linked to cancer, and not all cancers are caused by DNA viruses, EBV, KSHV, HPV, HBV, and MCPyV are examples of how the infectious cycles of DNA viruses create vulnerabilities and increase host susceptibility to cancer development through the manipulation of important signaling proteins like Raf, MEK, and ERK [206,207].

Interestingly, protein kinases now represent one of the largest group of cellular targets in pharmaceutical drug development, particularly in newly synthesized oncology drugs [209]. Since the first kinase inhibitor was approved by the FDA in 2001, over 25 different small molecule kinase inhibitors have been approved for use [210]. The Raf inhibitors vemurafenib [211] and sorafenib [212] and MEK inhibitors trametinib [213] and selumetinib [214] represent efforts of drug discovery teams to highlight the clinical importance of protein kinases not only in the single-cell environment but to improve human health. These kinase inhibitors, originally developed to treat multiple types of human cancers including metastatic melanomas [210] and renal and hepatocellular carcinomas [215,216], signify an intriguing opportunity for virology researchers and clinicians to explore the impacts of these FDA-approved MAPK inhibitors on DNA virus infection. The complexity of biological signaling networks cannot be overstated, even in a canonical setting. These pathways are the interpreters of the cellular environment; one pathway can transmit many different signals, or one stimulus can be transmitted by many different pathways. In the cases of virus dysregulation of signaling pathways, these potentially redundant functions allow for multiple opportunities for successful infection. Further insight into the complexity of signaling pathway communication is required to gain a better understanding the cell itself and to clarify avenues that may be exploited by viruses and other pathogens.

## Figures and Tables

**Table 1:** Role of MAPK-ERK in infectious lifecycle of DNA viruses. (+) indicate viral induction of the MAPK-ERK pathway to facilitate the indicated response, with the associated viral protein that influences MAPK-ERK signaling indicated (if known).

Viral Family	Virus	MAPK-ERK Role in Infectious Lifecycle			
		Enhances Viral Replication	Cell Cycle Dysregulation	Altered Immune Response	Promote Cell Survival
*Adenoviridae*	HAdV	+		+	
		(E4-ORF1)			
*Poxviridae*	VACV	+			
		(O1)			
*Polyomaviridae*	JCPyV	+			
		(tAg)			
	BKPyV	+	+		
			(tAg)		
*Papillomaviridae*	HPV	+			+
					(E5)
*Herpesviridae*	HSV-1	+	+	+	+
			(ICP0)	(ICP34.5)	(US3)
	HSV-2	+	+		+
			(ICP10)		(ICP10)
	KSHV	+			
		(ORF45)			
	EBV	+			
		(LMP1/2A)			
*Hepadnaviridae*	HBV	+	+	+	
		(HBx)	(MHBs(t))	(HBcAg)	
